# BMC family practice integrated GP care for patients with persistent physical symptoms: feasibility cluster randomised trial

**DOI:** 10.1186/s12875-020-01269-9

**Published:** 2020-10-07

**Authors:** Meenal Patel, Kirsty James, Rona Moss-Morris, Mark Ashworth, Mujtaba Husain, Matthew Hotopf, Anthony S. David, Paul McCrone, Sabine Landau, Trudie Chalder, on behalf of the PRINCE Primary trial team, Nicola Ferreira, Katie Watts, Richard Turner, Alisia Carnemolla, Jennifer Robertson, Shinal Patel, Philipp Frank, Paige Fisher-Smith, Abigale Childs, Iris Mosweu, Claire Willis, Fabio Simiao

**Affiliations:** 1 Department of Psychological Medicine, Institute of Psychiatry, Psychology and Neuroscience, King’s College London, 16 De Crespigny Park, London, SE5 8AF UK; 2 Department of Biostatistics and Health Informatics, Institute of Psychiatry, Psychology and Neurosciences, Psychology and Neuroscience King’s College, London, UK; 3 Psychology Department, Institute of Psychiatry, Psychology and Neuroscience, King’s College, London, UK; 4 School of Population Health and Environmental Sciences, Faculty of Life Sciences and Medicine King’s College London, London, UK; 5 UK South London and Maudsley NHS Foundation Trust, London, UK; 6 Division of Psychiatry, Maple House, UCL Institute of Mental Health, 149 Tottenham Court Road, London, W1T 7NF UK; 7 Institute for Lifecourse Development, University of Greenwich, Old Royal Naval College, Park Row, Greenwich, London, SE10 9LS UK

**Keywords:** Persistent physical symptoms, Cognitive behavioural skills, Feasibility, General practice, Cluster randomised controlled trial, Transdiagnostic

## Abstract

**Background:**

Patients continue to suffer from medically unexplained symptoms otherwise referred to as persistent physical symptoms (PPS). General practitioners (GPs) play a key role in the management of PPS and require further training. Patients are often frustrated with the care they receive. This study aims to assess the acceptability of an ‘integrated GP care’ approach which consists of offering self-help materials to patients with PPS and offering their GPs training on how to utilise cognitive behavioural skills within their consultations, as well as assessing the feasibility of conducting a future trial in primary care to evaluate its benefit.

**Methods:**

A feasibility cluster randomised controlled trial was conducted in primary care, South London, UK. GP practices (clusters) were randomly allocated to ‘integrated GP care plus treatment as usual’ or ‘treatment as usual’. Patients with PPS were recruited from participating GP practices before randomisation. Feasibility parameters, process variables and potential outcome measures were collected at pre-randomisation and at 12- and 24-weeks post-randomisation at cluster and individual participant level.

**Results:**

Two thousand nine hundred seventy-eight patients were identified from 18 GP practices. Out of the 424 patients who responded with interest in the study, 164 fully met the eligibility criteria. One hundred sixty-one patients provided baseline data before cluster randomisation and therefore were able to participate in the study. Most feasibility parameters indicated that the intervention was acceptable and a future trial feasible. 50 GPs from 8 GP practices (randomised to intervention) attended the offer of training and provided positive feedback. Scores in GP knowledge and confidence increased post-training. Follow-up rate of patients at 24 weeks was 87%. However estimated effect sizes on potential clinical outcomes were small.

**Conclusions:**

It was feasible to identify and recruit patients with PPS. Retention rates of participants up to 24 weeks were high. A wide range of health services were used. The intervention was relatively low cost and low risk. This complex intervention should be further developed to improve patients’/GPs’ utilisation of audio/visual and training resources before proceeding to a full trial evaluation.

**Trial registration:**

NCT02444520 (ClinicalTrials.gov).

## Background

Medically unexplained symptoms (MUS), otherwise referred to as persistent physical symptoms (PPS) [1, 2], cannot be explained by organic pathology after medical examinations [3, 4]. The prevalence of PPS in primary care is high, ranging between approximately 11 and 65% [5–7]. Furthermore, depending on severity and circumstance PPS can be linked to functional impairment, psychological distress as well as being expensive to the healthcare system [3, 8]. Although, clinician uncertainty in diagnosing these symptoms, often leads to over investigation and unnecessary treatments [9, 10], a recent study found that GPs did not experience clinical uncertainty. They were more likely to provide a diagnosis of PPS if a) they were cognizant of the patient’s medical and social history, b) a discrepancy between the symptoms presented by the patient and objective findings were apparent c) patients reported several symptoms and lacked clarity about the nature of their symptoms [11].

Patients with PPS tend to feel dissatisfied with the care they receive and often feel misunderstood [12, 13]. Although they can be referred to specialist services, referrals are often associated with barriers such as geographical restrictions, costs and stigma [14, 15]. GPs play a key role in managing patients with PPS. However, GPs often feel powerless to influence patients’ understanding of their illness [4, 9, 16], despite understanding the importance of good communication in terms of exploring psychosocial cues and engaging in a more patient centred approach [17].

There is evidence to suggest that psychological therapies, including cognitive behavioural therapy (CBT), can be used to change the way patients perceive their symptoms in order to help manage them more effectively. A systematic review published by van Dessel et al. (2014) [18] assessed the effects of non-pharmacological interventions for somatoform disorders and concluded that studies comparing some form of psychological therapy to treatment as usual or a waiting list resulted in less severe symptoms at the end of treatment. Twenty-one studies were included, however effects sizes were considered small [18]. In addition, most of the studies in this review used forms of enhanced care (based broadly on a bio-psycho-social model that encouraged patients to develop strategies for dealing with their physical symptoms) as the control treatment. This made it hard to assess the effectiveness of the psychological therapy being evaluated.

A systematic review published by Rosendal et al. (2013) [19] assessed the clinical effectiveness of enhanced care interventions for adults with functional somatic symptoms in primary care. Six RCT’s were included which were heterogeneous in terms of trial design, patient populations, intervention characteristics and outcome measures. The authors concluded that the review could not answer whether enhanced care had an effect or not on outcomes. The studies only intervened at GP level. This suggests that if a more integrated approach was adopted, i.e. both GPs and patients were provided with resources, there may be an effect [19].

A systematic review and meta-analysis published by van Gils et al. (2013) concluded that self-help (designed to be conducted independently from healthcare workers) compared with usual care or waiting list, was associated with lower symptom severity and higher QoL directly post treatment, irrespective of amount of therapist content for adults with MUS [20].

Although PPS are many and varied, there are a range of common responses patients with PPS have to these symptoms, including avoidance of activity, poor sleep routines and catastrophising [21]. Transdiagnostic theory suggests that by targeting these common processes the same treatment can be used across different symptom clusters as long as flexibility is used with each individual patient [22].

Given the high prevalence of PPS in primary care, the fact that symptoms tend to cluster together and the pressure to manage a range of different symptoms during a short consultation, a transdiagnostic approach may be suitable in primary care. However, this requires the GP to be skilled in describing the approach and patient willingness to engage in behaviour change.

Taking these previous findings into account, we developed an ‘integrated GP care’ approach. by providing self-help materials to patients and offering GP practices (clusters) training on how their GPs could utilise behavioural skills within short consultations [21].

The aim of this study was to assess the acceptability of the approach and the feasibility of conducting a future trial within the context of a cluster randomised controlled trial in primary care. This trial compared ‘integrated GP care plus treatment as usual’ to treatment as usual.

## Methods

The trial design has been described in a published protocol [23]. Here we provide a brief summary.

### Study design and participants

A two-arm cluster randomised, waiting list-controlled, trial conducted in South London, UK: Outcome data were collected pre-randomisation and at 12- and 24-weeks post-randomisation. ‘Integrated GP care plus treatment as usual (TAU)’ approach or TAU were randomly allocated to GP practices (clusters). Patients were recruited into the trial before randomisation of their cluster. Consent from each GP practice cluster was provided by either the practice manager/lead GP. Eligibility criteria for GP practices and patients are outlined in Table 1. Patients were recruited between August 2015 and June 2017 from eligible GP practices and were identified via a search algorithm using a database, EMIS Web (the clinical software supporting electronic health records), commonly used by general practices in the UK. Every clinical term within EMIS Web is uniquely referred to as a ‘Read Code’. The bespoke search algorithm incorporated a range of Read Codes that were related to PPS including specific and widespread back pain, chronic/multiple widespread pain, dizziness, fatigue, fibromyalgia, headaches, non-cardiac chest pain and medically unexplained symptom. The search algorithm also included elements of the eligibility criteria. Identified patients were sent a letter by their GP practice and were asked to respond to the research team to say whether they were interested/not interested in participating [23]. Patients who gave consent received a telephone call from the research team to ensure that the patient eligibility criteria into the study was fully met (see Table 1). If the research team were not confident of the diagnosis, the patients GP practice was contacted for clarification. GP practices included a file note via EMIS Web for each participant to remind GPs which patients had consented to participate in the study. All participants recruited provided individual consent and thereafter were asked to complete the baseline assessments prior to randomisation.

**Table 1:** GP Practice and Patient Eligibility Criteria

	Inclusion Criteria	Exclusion Criteria
GP Practices (clusters)	Situated in South London, UK	Risk of closure
	The lead GP or other authorised individual provided consent for the GP practice to take part in the study	
	At least 50% of GPs within the GP practice were interested in completing the training workshop	
Patients	A PPS diagnosis	Active psychosis
	≥18 years old, ≤65 years old	Drug or alcohol addiction
	Registered within a GP practice in South London that has consented to taking part in PRINCE Primary	Benzodiazepine use exceeding 10 mg per day
	Had 6 or more consultations in the last year (not necessarily for the same symptom or directly related to PPS)	Had any psychotherapy treatment within the last year for their PPS
	Ability to give written informed consent	Dissociative seizures^a^
	Provided baseline data	Imminent risk of self-harm
	Speak and read English at an adequate level	Taking part in PRINCE Secondary (NCT02426788) or the ACTIB study (ISRCTN44427879)

^a^ due to an ongoing RCT at time of recruitment that was evaluating a specific cognitive behavioural approach for Dissociative Seizures, now published [24]

Randomisation of GP practices occurred on a pre-specified date. Practices participating in the trial were willing to ‘pencil in’ a date in advance with the knowledge that they may not receive the offer of training. Participants of GP practices randomised to ‘integrated GP care plus TAU’ were sent the self-help material within 1 week of randomisation. GP’s received the training within 2 weeks of randomisation. The randomisation date was considered the anchor point for all trial participants and subsequent measures were taken at 12 and 24 weeks.

### Randomisation and blinding

Randomisation of GP practice clusters was carried out after recruitment and baseline assessments of patients within each GP practice. Cluster (i.e. GP practice) randomisation was stratified by size of GP practice (≤ 6000 registered patients or > 6000 registered patients) and was coordinated by an independent randomisation service at the UKCRC registered King’s Clinical Trials Unit (KCTU).

Outcome assessors were blind to treatment allocation. The junior trial statistician was unblinded during the trial; the senior trial statistician and chief investigator remained partially blind (i.e. only knew groups as A and B) until the final stages of the analysis.

### Description of intervention and treatment as usual

GP practices were randomised to ‘integrated GP care plus TAU’ or ‘TAU’. The intervention, ‘integrated GP care plus TAU’ is fully described in the published protocol [23] and summarised below.

The intervention involved both patients and GPs (see Table 2). The GP training (certified as ‘Continuing Professional Development’ (CPD)) was delivered by experienced clinicians based within King’s Health Partners. All GP’s opted for 90 min training. They were provided with a theoretical model of understanding “persistent physical symptoms” [3]. A distinction between the role of predisposing, precipitating and perpetuating factors associated with symptoms was made. We then focused on teaching cognitive behavioural skills we thought could realistically be utilised within a 10 min consultation. Specifically, we demonstrated via role-play the negotiation of patient centred, behavioural goals. GPs were provided with prompt sheets and could access other training material such as videos demonstrating skills via a website. We suggested GPs use the skills during routine practice and did not encourage extra consultations. We assessed knowledge and confidence pre and post training and satisfaction after training had been completed.

**Table 2:** Integrated GP care plus treatment as usual

Component	Brief Description of Intervention
GP Training	GPs practices were offered training in utilising cognitive behavioural skills during 10-min consultations.
GP Supervision	GP practices were offered additional GP training and supervision with either or both a Psychiatrist and therapist
Audio-visual and written materials/guidelines for GP’s	All GPs that attended the training were provided with study-specific guidance on how to change the nature of consultations. A list of helpful responses in the consultation with patients were provided. They also had access to a website which contained resources including role play demonstrations of skills and all resources that were given to participants in the trial.
Participants’ Booklets	A series of booklets were sent to participants’ homes, these included (i) an introduction to PPS, (ii) how to juggle activities, (iii) improving sleep, (iv) living with uncertainty, (v) emotional well-being and (vi) goal setting. Participants were also sent symptom booklets that included information about their primary symptoms.
Participants’ Animation	Participants had access to an animation describing a patient’s experience with chronic pain

We provided participants with self-help materials which targeted a range of key behavioural, emotional and cognitive processes. These included booklets on activity management, living with uncertainty, emotional wellbeing, goal setting and establishing a sleep routine. Information in the booklets dovetailed with information shared with GP’s during training to ensure a consistent message was being provided. Participants also had access to an animation which illustrated the approach, via a website specifically set–up for the trial. Participants in the TAU arm were given no additional resources.

### Feasibility parameters and potential outcome measures

The following parameters were estimated to assess whether it was feasible to conduct an adequately powered trial to evaluate the integrated GP care approach:

a Willingness of GP practices to be contacted about PRINCE Primary
b Willingness of GP practices to consent and be randomised
c Availability of data needed and the usefulness and limitations of the general practice databases
d Interest of patients to be contacted about the study
e Rate of eligible participants
f Willingness of patients to consent to participate in PRINCE Primary
g Willingness of participants to complete baseline measures before randomisation
h Interest of GPs to attend the GP training (intervention arm only)
i Participants follow-up rates to questionnaires per group

A number of potential outcome measures for use in a future trial were collected at baseline, 12- and 24-weeks post randomisation. Participants were asked to complete all measures and had the option to complete them via post, email or telephone. The measures were:

- Psychosocial Functioning: the Work and Social Adjustment Scale (WSAS) [25] was used to measure the impact of PPS on patients daily functioning in terms of work and home management, social and private leisure activities as well as close relationships.
- Physical Symptoms: the Patient Health Questionnaire 15 (PHQ-15) [26] was used to measure symptom severity.
- Psychological Distress: The Patient Health Questionnaire 9 (PHQ-9) [27] was used to measure the severity of depression.
- Global Outcome: the adapted Clinical Global Impression (CGI) [28] was used to measure global change.
- Cost Effectiveness (Service) and health benefits: health service use (including hospital attendances and admissions, GP contacts), informal care, lost work time and financial benefits was measured using the an adapted Client Service Receipt Inventory [29] and the EQ-5D [30, 31]

Process measure:

- The Cognitive Behavioural Responses Questionnaire (CBRQ) [32] was used to assess potential mechanisms of change. The questionnaire is formed of 5 subscales which includes fear avoidance, catastrophising, damage avoidance, embarrassment avoidance and symptom focussing.

All measures have been shown to have good reliability and validity. If participants did not complete follow-up measures, the research assistant contacted them to remind them that they were due to be completed.

GPs’ knowledge, confidence and satisfaction: GPs who attended training were asked to complete knowledge and confidence self-report measures pre and post training. *GP knowledge*: This encompassed 10 true/false questions relating to the content of the training. *GP Confidence*: questions asked how confident they were at dealing with patients with PPS. Items ranged from 1 to 7 where 1 was not at all confident to 7 very confident.

Post training only: GPs were asked to provide immediate feedback post-training using a self-report measure. Questions related to (i) general content (ii) relevance to their job (iii) learning material (iv) the instructor and (v) overall satisfaction of the training. For these subscales the items ranged from 1 to 7 where 1 was strongly disagree to 7 which was strongly agree. In addition, GPs were asked whether they found specific aspects of the training (eg. setting agendas, negotiating behavioural skills and activity scheduling etc) helpful or not. Items ranged from 1 to 7 where 1 was very unhelpful to 7 which was very helpful.

### Statistical analysis

We planned to recruit a selection of 16–20 GP practices from the London boroughs of Lambeth and Southwark with an expected patient sample size of 240 (see protocol [23]). As the trial progressed the recruitment area was expanded to other South London regions to increase recruitment.

Each feasibility parameter corresponds to a letter (please see ‘*Feasibility Parameters and Potential Outcome Measure*’. Feasibility parameter (h) was quantified by comparing the number of GPs registered at a practice to the number who attended training. Feasibility parameters (a), (b), (d) – (g) and (i) were estimated using information from the Consolidation Standards of Reporting Trials (CONSORT) diagram. Potential outcomes for a future trial were summarised by arm at baseline and at each follow-up time point. Means/standard deviations or medians/ranges were calculated depending on the distribution of the measure. In addition, for continuous outcome measures which might serve as the primary outcome intra cluster (GP practice) correlation coefficients (ICC) were calculated.

Inferential analyses were carried out to estimate intervention effects for potential outcome variables. These analyses estimated the differences in mean outcomes between patients in practices randomised to integrated GP care and those in control practices by intention to treat. Trial arm differences and associated 95% confidence intervals were generated based on linear mixed models fitted using maximum likelihood. These mixed models contained post-treatment measures of the outcome at 12 and 24 weeks as the dependent variable. Fixed effects consisted of: baseline measures of the outcome, trial arm, randomisation stratifier, a dummy variable for time point and a trial arm x time interaction term. A participant-varying random intercept accounted for correlations between the two repeated measures per participant and a GP practice-varying random intercept accounted for correlations between patients within the same practice. This study was a feasibility study and therefore no formal significance tests were carried out. The effect size was standardised by dividing the estimated mean difference by the respective standard deviation at baseline.

*Health Economics:* No formal statistical analysis was completed on the CSRI and EQ-5D as this was a feasibility trial. Instead descriptive data is reported on the numbers and percentages, means and standard deviations of using these services in the last 6 months. Information is also provided on lost work due to illness.

### Patient and public involvement

Patient and public involvement (PPI) representatives were included in all phases of the study design, to ensure that the trial was not burdensome for patients and realistic for GP’s. A PPI representative was involved in the Trial Steering Committee and offered comments on aspects of the research.

## Results

### Feasibility of recruitment and retention

**Fig. 1:**
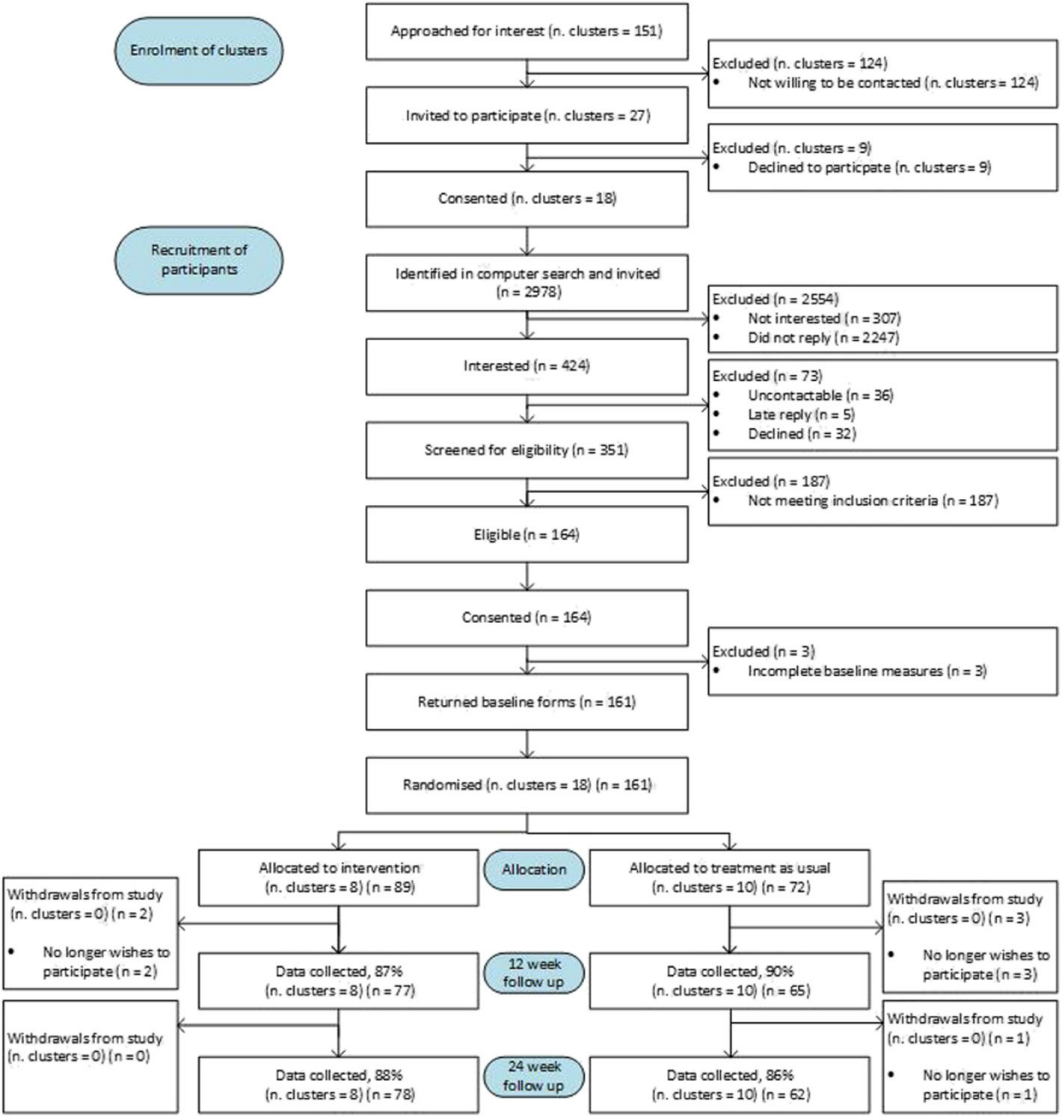
Consolidation Standards of Reporting Trials (CONSORT) diagram for Persistent Physical Symptoms Reduction Intervention in Primary Care (PRINCE) Trial

The CONSORT diagram (see Fig. 1) describes GP practices and participants’ journey. One hundred fifty-one GP Practices (clusters) were approached for interest. (a) 27/151 (18%) GP practices were willing to be contacted. (b) Of these 18/27 (67%) GP practices consented to participate. Of these 8 were later randomised to the intervention group and 10 to TAU. (c) From GP practices that consented, 2978/193,824 (2%) of registered patients were identified as potentially eligible. (d) Of those identified 731/2978 (25%) patients replied with either an interest (*n* = 424) or no interest (*n* = 307). (e) From this cohort, 164/424 patients (39%) met inclusion criteria, (f) 100% consented to participate (g) of those consenting 161 (98%) completed baseline measures before cluster randomisation took place. Of these, 89 were from GP practices later randomised to the intervention and 72 from GP practices allocated to TAU. (i) Questionnaires were returned by 142 (88%) participants at 12 weeks and 140 (87%) participants at 24 weeks.

Estimates of feasibility parameters and associated confidence intervals are summarised in Table 3.

**Table 3:** Feasibility parameter estimates and 95% confidence intervals

Feasibility parameter	Proportion *%* [CI]
a. Willingness of GP practices to be contacted about the study *(number of GP practices responding out of those approached)*	18 [12, 24]
b. Willingness of practices to consent and be randomised *(number consenting out of those that were interested)*	67 [46, 83]
c. Availability of data needed and the usefulness and limitations of the general practice databases (number of patients identified using GP informatics (search algorithm) out of patients registered with the GP practice).	2 [1, 2]
d. Interest of patients to be contacted about the study *(Number of patients responding out of patients identified in search)*	25 [23, 26]
e. Rate of eligible participants *(Number of patients meeting eligibility criteria out of those interested)*	39 [34, 43]
f. Willingness of patients to consent *(number consenting out of number eligible)*	100
g. Willingness of participants to complete baseline measures before randomisation *(number sending back baseline forms out of number consented)*	98 [95, 99]
h. Interest of GPs to attend the GP training (intervention arm only)	N/A
i. Participants follow up rates to questionnaires *(number of participants completing packs out of those randomised)*	
*12 weeks*	88 [82, 93]
*24 weeks*	87 [81, 92]

260 (61%) patients who responded with an interest towards the study were excluded; 73 (28%) patients were not screened (uncontactable/declined/late response) and 187 (72%) did not meet the eligibility criteria. During screening 43% (81/187) of patients were identified as having a medical diagnosis linked to their symptoms. For example, a patient may have been identified through a read code as having back pain, however this may be due to a slipped disc and therefore the pain was explained.

### Baseline characteristics

*GP Practices/GPs:* GP practices had an average of 6 GPs registered with them at the time of consent. Mean number of patients registered in a practice was 10,768 (SD = 2966.5; Range = 5571–17,489). 102 GPs from 17 GP practices provided demographic information. There was a reasonable balance between trial arms in GP characteristics, but GPs in the intervention arm were slightly older (42 years; SD = 11.9 Range = 24–63 v 38 years; SD = 9.3 Range 23–57) and more likely to be male (49% v 38.5%, respectively) than GPs in the control group.

#### Participants

Table 4 shows participants’ baseline demographic and clinical characteristics. Most participants were female. 22% had previous experience of CBT. Participants reported having significant but not severe functional impairment (WSAS) [25]. The mean PHQ-15 score was 13.6 (*N* = 157; SD = 5.9), suggesting moderate levels of symptom severity [33]. The mean PHQ-9 score was 9.8 (*N* = 160; SD = 6.5) with 48% scoring above the clinical cut-off for moderate depression (PHQ-9 > 9) [27]. Widespread pain (21%), back pain (20%) and fatigue (16%) were the most prevalent symptoms.

**Table 4:** Participants demographic and clinical characteristics of the study sample at baseline (*n* = 161)

		Intervention *n* = 89	Control *n* = 72	Overall *n* = 161
Age	Mean (SD)	48.4 (11.7)	44.2 (11.0)	46.5 (11.6)
Sex	Female (%)	70 (78.7)	60 (83.3)	130 (80.7)
Ethnicity	White (%)	46 (51.7)	37 (51.4)	83 (51.6)
First Language	English (%)	71 (79.8)	56 (77.8)	127 (78.9)
Relationship status	With Partner (%)	37 (41.6)	34 (47.2)	71 (44.1)
Cohabitation status	Alone (%)	22 (24.7)	11 (15.3)	33 (20.5)
Children	Yes (%)	52 (58.4)	40 (55.6)	92 (57.1)
Dependent elderly relatives	Yes (%)	14 (15.7)	11 (15.3)	25 (15.5)
Accommodation status (%)	Owner occupied flat / house	31 (34.8)	15 (20.8)	46 (28.6)
	Privately rented flat / house	26 (29.2)	23 (31.9)	49 (30.4)
	Flat / house rented from local authority	23 (25.8)	27 (37.5)	50 (31.1)
	Other	8 (9.0)	7 (9.7)	15 (9.3)
	Missing	1 (1.1)	0	1 (0.6)
Highest level of education *(%)*	No GCSE or equivalent	6 (6.7)	5 (6.9)	11 (6.8)
	GCSE / O level or equivalent	23 (25.8)	19 (26.4)	42 (26.1)
	A level or equivalent	13 (14.6)	11 (15.3)	24 (14.9)
	Degree	21 (23.6)	19 (26.4)	40 (24.8)
	Postgraduate	16 (18.0)	7 (9.7)	23 (14.3)
	Other	9 (10.1)	10 (13.9)	19 (11.8)
	Missing	1 (1.1)	1 (1.4)	2 (1.2)
Previous receipt of CBT	Yes (%)	19 (21.3)	17 (23.6)	36 (22.4)
Previous receipt of physiotherapy	Yes (%)	63 (70.8)	56 (77.8)	119 (73.9)
Previous receipt of other therapy	Yes (%)	31 (34.8)	28 (38.9)	59 (36.6)
PPS Subtype^a^ n (%)	Overall Pain	26 (21.0)	24 (20.3)	50 (20.7)
	Fibromyalgia	5 (4.0)	11 (9.3)	16 (6.6)
	IBS	20 (16.1)	15 (12.7)	35 (14.5)
	Dizziness	5 (4.0)	8 (6.8)	13 (5.4)
	Back Pain	29 (23.4)	20 (16.9)	49 (20.2)
	Fatigue	23 (18.7)	15 (12.7)	38 (15.8)
	Headache	10 (8.1)	19 (16.1)	29 (12.0)
	Non-Cardiac Chest Pain	1 (0.8)	1 (0.8)	2 (0.8)
	Limb Weakness	3 (2.4)	3 (2.5)	6 (2.5)
	Shortness of Breath	2 (1.6)	1 (0.8)	3 (1.2)
	POTS	0 (0.0)	1 (0.8)	1 (0.4)
WSAS	N	87	72	161
	Mean (SD) [range]	19.7 (10.8) [0.0, 40.0]	19.0 (11.3) [0.0, 40.0]	19.4 (11.0) [0.0, 40.0]
PHQ 15	N	86	71	157
	Mean (SD) [range]	13.2 (5.5) [3.0, 26.8]	14.1 (6.4) [3.0, 27.9]	13.6 (5.9) [3.0, 27.9]
PHQ 9	N	88	72	160
	Mean (SD) [range]	9.9 (6.4) [0.0, 25.0]	9.8 (6.8) [0.0, 26.0]	9.8 (6.5) [0.0, 26.0]
Below cut off	n (%)	18 (20.5)	21 (29.2)	39 (24.4)
Mild	n (%)	32 (36.4)	13 (18.1)	45 (28.1)
Moderate	n (%)	17 (19.3)	23 (31.9)	40 (25.0)
Moderately severe	n (%)	13 (14.8)	8 (11.1)	21 (13.1)
Severe	n (%)	8 (9.1)	7 (9.7)	15 (9.4)
CBRQ				
*Catastrophising*	N	89	71	160
	Mean (SD) [range]	9.9 (3.4) [0.0, 16.0]	9.5 (3.4) [1.0, 16.0]	9.7 (3.4) [0.0, 16.0]
*Fear avoidance*	N	89	71	160
	Mean (SD) [range]	10.7 (4.4) [1.0, 24.0]	11.1 (4.8) [0.0, 20.0]	10.9 (4.5) [0.0, 24.0]
*Embarrassment avoidance*	N	89	70	159
	Mean (SD) [range]	10.9 (6.4) [0.0, 24.0]	10.1 (7.0) [0.0, 24.0]	10.5 (6.6) [0.0, 24.0]
*Damage*	N	88	69	157
	Mean (SD) [range]	11.1 (2.4) [6.0, 18.0]	11.3 (3.3) [4.0, 18.0]	11.2 (2.8) [4.0, 18.0]
*Symptoms*	N	89	70	159
	Mean (SD) [range]	14.7 (5.0) [3.0, 28.0]	14.9 (6.0) [0.0, 28.0]	14.8 (5.5) [0.0, 28.0]

^a^Participants may have reported more than one PPS. IBS Irritable Bowel Syndrome WSAS Work and Social Adjustment Scale, PHQ-15 Patient Health Questionnaire 15, PHQ 9 Patient Health Questionnaire 9, CBRQ Cognitive Behavioural Responses Questionnaire

Participants completed a questionnaire on symptom attribution [34, 35] in which 66/156 (42%) reported that their symptoms were both physical/psychological in nature. 87/156 (56%) reported their symptoms were either physical or mainly physical and 3/156 (2%) reported their symptoms were psychological/mainly psychological in nature. Baseline characteristics were well balanced between the groups.

### GP training

One of the feasibility parameters (h) was to provide information on the uptake of the training by GPs. However, there were more GPs attending the training than were reported as working at the practice at randomisation, due to the inclusion of GP trainees and locums.

*GPs knowledge, confidence and satisfaction:* 50 GPs attended training. There was an improvement in knowledge (median score = 10), but this was high pre-training (median = 9). In all domains, confidence increased between pre and post training. GPs gave positive feedback post-training with all items having a median response of 6 with 7 being the most positive response. No GP practice requested additional training/supervision.

### Identification of potential primary and secondary outcome measures

Potential patient outcome measures are summarised by trial arm and time-point (12 or 24 weeks) in Table 5.

**Table 5:** Summaries of outcome variables by trial arm

Follow-up time point		12 weeks			24 weeks		
Clinical scale		Intervention ***n*** = 89	Control ***n*** = 72	Overall ***n*** = 161	Intervention ***n*** = 89	Control ***n*** = 72	Overall ***n*** = 161
**WSAS**	***N***	75	66	141	78	62	140
	***mean (sd) [range]***	18.3 (11.5) [0.0, 40.0]	18.4 (11.0) [0.0, 39.0]	18.3 (11.3) [0.0, 40.0]	17.9 (11.7) [0.0, 40.0]	17.2 (12.6) [0.0, 40.0]	17.6 (12.0) [0.0, 40.0]
**PHQ 15**	***N***	74	63	137	77	62	139
	***mean (sd) [range]***	13.2 (5.9) [2.0, 26.5]	13.7 (5.6) [3.0, 27.0]	13.4 (5.8) [2.0, 27.0]	12.7 (5.7) [1.0, 27.7]	13.1 (5.6) [1.0, 23.1]	12.9 (5.7) [1.0, 27.7]
**PHQ 9**	***N***	75	64	139	78	62	140
	***mean (sd) [range]***	9.1 (6.4) [0.0, 25.0]	9.5 (6.2) [0.0, 26.0]	9.3 (6.3) [0.0, 26.0]	9.2 (6.4) [0.0, 27.0]	8.1 (5.9) [0.0, 24.0]	8.7 (6.2) [0.0, 27.0]
**Below cut off**	***n (%)***	19 (25.3)	16 (25.0)	35 (25.2)	21 (26.9)	22 (35.5)	43 (30.7)
**Mild**	***n (%)***	25 (33.3)	18 (28.1)	43 (30.9)	25 (32.1)	17 (27.4)	42 (30.0)
**Moderate**	***n (%)***	14 (18.7)	17 (26.6)	31 (22.3)	17 (21.8)	15 (24.2)	32 (22.9)
**Moderately severe**	***n (%)***	11 (14.7)	8 (12.5)	19 (13.7)	9 (11.5)	5 (8.1)	14 (10.0)
**Severe**	***n (%)***	6 (8.0)	5 (7.8)	11 (7.9)	6 (7.7)	3 (4.8)	9 (6.4)
**CGI**	***N***	76	67	143	79	63	142
	***mean (sd) [range]***	4.9 (1.3) [2.0, 9.0]	4.8 (1.4) [1.0, 9.0]	4.8 (1.4) [1.0, 9.0]	4.8 (1.5) [2.0, 9.0]	4.7 (1.5) [2.0, 9.0]	4.8 (1.5) [2.0, 9.0]
**CBRQ**							
**Catastrophising**	***N***	77	65	142	78	61	139
	***mean (sd) [range]***	8.7 (3.9) [0.0, 16.0]	9.4 (3.1) [3.0, 16.0]	9.0 (3.6) [0.0, 16.0]	8.6 (4.0) [0.0, 16.0]	8.8 (3.7) [0.0, 16.0]	8.7 (3.8) [0.0, 16.0]
**Fear avoidance**	***N***	77	65	142	76	59	135
	***mean (sd) [range]***	10.4 (4.7) [0.0, 24.0]	10.6 (5.1) [0.0, 24.0]	10.5 (4.9) [0.0, 24.0]	10.3 (5.0) [1.0, 24.0]	10.1 (4.6) [1.0, 20.4]	10.2 (4.8) [1.0, 24.0]
**Embarrassment avoidance**	***N***	77	65	142	78	61	139
	***mean (sd) [range]***	10.5 (6.6) [0.0, 24.0]	10.7 (6.9) [0.0, 24.0]	10.6 (6.7) [0.0, 24.0]	10.3 (6.1) [0.0, 24.0]	9.7 (7.0) [0.0, 24.0]	10.0 (6.5) [0.0, 24.0]
**Damage**	***N***	76	65	141	76	59	135
	***mean (sd) [range]***	10.1 (3.0) [4.0, 20.0]	10.6 (3.5) [2.0, 18.0]	10.3 (3.2) [2.0, 20.0]	9.8 (3.0) [1.0, 16.0]	10.2 (3.4) [4.0, 18.0]	10.0 (3.2) [1.0, 18.0]
**Symptom Focusing**	***N***	77	65	142	77	61	138
	***mean (sd) [range]***	13.8 (6.2) [0.0, 27.0]	14.5 (5.4) [2.0, 28.0]	14.1 (5.8) [0.0, 28.0]	13.5 (5.7) [2.0, 28.0]	13.5 (5.1) [2.0, 26.0]	13.5 (5.4) [2.0, 28.0]

WSAS Work and Social Adjustment Scale, PHQ-15 Patient Health Questionnaire 15, PHQ 9 Patient Health Questionnaire 9, CGI Clinical Global Impression, CBRQ Cognitive Behavioural Responses Questionnaire

Estimates of trial arm differences in potential continuous outcome measures of a future trial were calculated. None of the outcomes provided evidence for trial arm differences; all estimated effect sizes were small. Largest effect sizes in favour of the intervention were estimated for CBRQ-Catastrophising (0.11) and CBRQ- Damage beliefs (0.23) at 24 weeks.

To aid the planning of any future trial we estimated the intra-cluster (GP practice) correlation coefficients (ICC). While we estimated ICCs in the range from 0.05 to 0.16 at baseline no such correlations could be found for the 24-week continuous outcomes adjusted for baseline effects. This suggests that cluster effects are minimal under the current trial design if baseline values are measured and accounted for in any modelling.

### Health economics

Table 6 shows the use of services in each group at baseline and at 24-week follow-up. At baseline and follow-up the most commonly used services were general practitioners, other doctors, practice nurses, pharmacists, physiotherapists, and alternative therapists (providing interventions such as acupuncture, aromatherapy, etc). Relatively little use was made of occupational therapists and social workers. Inpatient care was used by 10% or less of the sample. The most commonly used tests were MRIs, X-rays, ultrasounds and blood tests. Informal care from family members or friends was received by a high proportion of participants, particularly for help in and outside the home. Finally, lost production was experienced by around one-third of each group at both baseline and follow-up.

**Table 6:** Use of health services by trial arm

		Intervention		Control	
	Measure	Baseline (***n*** = 89)	24-week (***n*** = 79)	Baseline (***n*** = 72)	24-week (***n*** = 62)
Community services					
General practitioner	Contact	82 (92)	67 (85)	66 (92)	53 (85)
Psychiatrist	Contact	2 (2)	4 (5)	3 (4)	2 (3)
Other doctor	Contact	37 (42)	40 (51)	34 (47)	29 (47)
Practice nurse	Contact	33 (37)	28 (35)	25 (35)	17 (27)
Pharmacist	Contact	31 (35)	26 (33)	25 (35)	18 (29)
Physiotherapist	Contact	28 (43)	24 (30)	23 (32)	14 (23)
Social worker	Contact	2 (2)	1 (1)	1 (1)	3 (5)
Psychologist/therapist	Contact	9 (10)	14 (18)	10 (14)	9 (15)
Community mental health worker	Contact	1 (1)	3 (4)	1 (1)	0
Alternative treatment	Contact	23 (26)	19 (24)	17 (24)	15 (24)
Occupational therapist	Contact	5 (6)	5 (7)	9 (13)	3 (5)
Hospital-based services					
Inpatient	Length of stay	9 (10)	7 (9)	7 (10)	5 (8)
Outpatient	Contact	17 (19)	12 (15)	16 (22)	10 (16)
Tests					
MRI	Contact	22 (25)	15 (19)	16 (22)	10 (16)
CT/CAT scan	Contact	11 (12)	8 (10)	11 (15)	8 (13)
Ultrasound	Contact	27 (30)	20 (26)	16 (22)	13 (21)
X-ray	Contact	34 (38)	23 (29)	30 (42)	16 (26)
EEG	Contact	7 (8)	6 (8)	6 (8)	3 (5)
Blood test	Contact	61 (69)	53 (67)	56 (78)	32 (52)
*Informal care*					
Personal care	Hours/Week	14 (16)	4 (5)	15 (21)	8 (13)
Child Care	Hours/Week	8 (9)	12 (15)	9 (13)	12 (19)
Help in home	Hours/Week	34 (38)	27 (34)	31 (43)	25 (40)
Help outside home	Hours/Week	30 (34)	22 (28)	29 (40)	21 (34)
Productivity loss					
Days off work due to ill health	Days	33 (37)	23 (30)	28 (39)	17 (28)
Hours off work due to ill health	Hours/week	34 (38)	23 (30)	28 (39)	17 (28)

Table 7 presents findings of the EQ-5D for the two groups at baseline and 24 week follow-up. Improvements were found for all dimensions in both groups at follow-up illustrated by the utility scores. Although the control group had a low mean utility score at baseline, at follow-up this was disproportionately higher.

**Table 7:** Use of health services by trial arm

EQ-5D health states & levels	Intervention		Control Group	
	Baseline	24 weeks	Baseline	24 weeks
	Participants N (%)	Participants N (%)	Participants N (%)	Participants N (%)
Mobility				
No problems in walking about	33 (37)	29 (37)	25 (35)	29 (47)
Slight problems in walking about	20 (25)	25 (32)	20 (28)	14 (23)
Moderate problems in walking about	18 (20)	15 (19)	11 (15)	11 (18)
Severe problems in walking about	18 (20)	8 (10)	15 (21)	7 (11)
Unable to walk about	0	1 (1)	0	1 (2)
Self-care				
No problems washing or dressing myself	57 (64)	51 (65)	44 (61)	46 (74)
Slight problems washing or dressing myself	16 (18)	17 (21)	10 (14)	6 (10)
Moderate problems washing or dressing myself	9 (10)	5 (6)	14 (19)	8 (13)
Severe problems washing or dressing myself	7 (8)	5 (6)	3 (4)	1 (2)
Unable to wash or dress myself	0	0	1 (1)	1 (2)
Usual activities				
No problems doing my usual activities	21 (24)	23 (29)	18 (25)	27 (44)
Slight problems doing my usual activities	32 (36)	23 (29)	21 (29)	15 (24)
Moderate problems doing my usual activities	19 (21)	25 (32)	15 (21)	12 (19)
Severe problems doing my usual activities	16 (18)	8 (10)	14 (19)	8 (13)
Unable to do my usual activities	1 (1)	0	4 (6)	0
Pain/discomfort				
No pain or discomfort	4 (4)	8 (10)	5 (7)	11 (18)
Slight pain or discomfort	22 (25)	20 (25)	14 (19)	17 (27)
Moderate pain or discomfort	34 (38)	31 (39)	22 (31)	18 (29)
Severe pain or discomfort	24 (27)	16 (20)	22 (31)	14 (23)
Extreme pain or discomfort	4 (4)	4 (5)	9 13)	2 (3)
Anxiety/depression				
Not anxious or depressed	25 (28)	21 (27)	28 (39)	23 (37)
Slightly anxious or depressed	29 (33)	31 (39)	17 (24)	20 (32)
Moderately anxious or depressed	20 (22)	17 (22)	13 (18)	15 (24)
Severely anxious or depressed	13 (18)	7 (9)	6 (8)	3 (5)
Extremely anxious or depressed	2 (2)	3 (4)	7 (10)	1 (2)
Utility score	0.62 (0.31)	0.67 (0.26)	0.59 (0.31)	0.71 (0.27)

### Withdrawals and adverse events

There were 6 withdrawals during the trial period. There were 4 serious adverse events recorded on 3 participants and 89 adverse events recorded on 53 participants with no event being considered as related to the intervention. The serious adverse events were mainly cardiovascular. The adverse events were mainly psychiatric. The distribution of types of events appeared similar in the two trial arms.

## Discussion

### Summary

We consider a future trial of the ‘Integrated GP Care’ feasible because the research parameters (see Table 1) that we assessed were met. However, the intervention needs to be optimised, possibly with therapist input, to bring about meaningful, enduring change in participant outcomes. GPs who attended training provided excellent feedback of the training but with no measure of GP utilisation of resources further work is required. A wide range of PPS were identified within our sample, with widespread pain (21%) being the most prevalent. The health economic analyses showed a wide range of services being used and the version of the CSRI used in the study would be appropriate in a full trial. Little use of occupational therapist and social workers occurred but where they were used they may have had benefit. Although inpatient care was received by few participants, this is an expensive service and so should be measured.

### Strengths and limitations

The study design had many strengths. We worked with GP’s to develop a systematic search to identify potential participants for the trial. This involved incorporating a range of persistent physical symptoms and elements of the eligibility criteria. This study used a search algorithm within EMIS Web which aimed to include all patients who might have been eligible. The targeted Read codes were agreed by GPs and specialists to ensure that all Read codes related to PPS were included. However, there is evidence to suggest that GPs vary in how they record consultations [36], and it is possible that the algorithm missed some individuals with PPS, as well as misclassifying some.

Although this method was time-efficient, recruitment of patients was relatively low and therefore further refinement of electronic patient searches is needed given that all patients who showed an interest gave consent if eligible. We anticipated that we would recruit approximately 20 patients from each GP practice given the high prevalence of PPS in primary care. We recruited participants via the post. Future studies should consider engaging GPs to recruit patients directly, although this could lead to recruitment bias.

Once consented, participation rates were good with only 6 (4%) participants withdrawing. The training was well received and had high participation, achieved by ensuring it was certified for Continuing Professional Development purposes. Confidence increased but general knowledge did not, as it was already high. A limitation of the feasibility study was that we were not able to directly observe GPs’ clinical practice. Although GPs were offered additional training/supervision this was not taken up.

A wealth of resources for the intervention were developed for both GPs and participants, however there were no indications to suggest that the effects for most outcomes could be considered clinically meaningful. Patients had no personal contact with a therapist and were left to read and understand the material that was sent to them via post independently. We did not record how the resources were utilised by participants. However, we conducted qualitative interviews to assess this in a proportion which we will publish on separately. This minimal intervention did not seem potentially effective and a more intensive intervention is required. This contradicts findings reported by van Gils et al. who concluded that self-help was associated with a significant reduction in symptom severity and improvement in quality of life for people with MUS [20].

### Comparison with existing literature

We compared our findings with those published by Burton et al. [37]. Burton et al. (2012) developed an intervention that included a structured set of consultations, delivered by one specifically trained GP with a specialist interest in PPS [37]. The GP used simple cognitive and behavioural techniques to help patients modify the impact the symptoms were having. This involved four appointments with the initial consultation lasting 1h, and therefore might not be practical in a real primary care setting. The results indicated that clinically significant benefits could potentially be achieved. However, the generalisability of the approach needed testing. Follow-up rates for the Burton trial and our trial were excellent at 12 weeks (84% v 88% respectively).

Unlike our study, Burton and colleagues showed that it was feasible to generate clinically significant benefits for patients with PPS. This was probably due to the intervention being delivered by one GP, face to face over several sessions. Our study used many GPs in different practices, with no pre-planned sessions. We therefore conclude that Burton’s findings did not generalise to large groups of GPs.

Although our study contacted many GP's it is possible that in our study GP’s who had an interest in PPS were more likely to take up the offer of training and that they already possessed good knowledge about the clinical problems in question. Salmon et al. (2007) reported that GPs who devalue their psychological skills are less likely to participate in training which may result in recruitment bias [38].

Most of the studies reviewed in Rosendal et al. (2013) [19] involved intensive training programs where reattribution was taught. Our trial tested the feasibility of a shorter training session with optional supervision. We gave GPs the option to access role-plays and prompt sheets after the training. Allowing GPs to review materials in their own time could help GPs who are not as confident in their own skills to engage [38]. Rosendal et al. (2013) highlighted a number of methodological limitations with the studies they reviewed, including recruitment bias. We attempted to address this issue by recruiting patients before the randomisation of GP practices. GPs may have been more likely to agree to participate prior to randomisation as our intervention only incorporated one training session.

## Conclusions

This feasibility trial identified a method for systematically identifying potential patients in primary care with PPS. The number of GP practices and patients recruited into the study were in keeping with other studies. We developed specific resources to help empower GPs and patients with PPS. Although GP's were willing to attend training, ways of enhancing skills of GPs requires further investigation. We carried out qualitative interviews to gain a better understanding of patients’ views about aspects of this study which we will report in a separate paper. Our low intensity intervention requires adaptation before we can test the efficacy in a fully powered randomised controlled trial. Given that some change in patients’ beliefs i.e. that symptoms signal tissue damage and catastrophising about impact of symptoms was observed, these may be targeted in future trials. Processes such as avoidance and all or nothing behaviour could be addressed via therapist input or a digital intervention with some guidance.

## Data Availability

Availability of data and materials: The datasets generated/analysed in this study will be anonymised and deposited in a repository at Kings College London. Bona-fide researchers can contact the corresponding author (TC) to use the data and materials but are required to clearly specify the research question a priori. No consent was provided for sharing data with third parties.

## Abbreviations

BABCP: British Association of Behavioural and Cognitive Psychotherapies
CBRQ: Cognitive Behavioural Responses Questionnaire
CBT: Cognitive Behavioural Therapy
CGI: Clinical Global Impression
CONSORT: Consolidation Standards of Reporting Trials
CPD: Continuing Professional Development
CSRI: Client Service Receipt Inventory
DMEC: Data Monitoring & Ethics Committee
EQ-5D: EuroQoL 5D
GP: General Practitioner
HTA: Health Technology Assessment
IAPT: Increasing Access to Psychological Therapies
IBS: Irritable Bowel Syndrome
ICC: Intra Cluster Correlation Coefficients
KCTU: King’s Clinical Trial Unit
MRC: Medical Research Council
MUS: Medically Unexplained Symptoms
N: Number
NHS: National Health Service
NIHR EME: National Institute of Health Research Efficacy and Mechanism Evaluation
PHQ-9: Patient Health Questionnaire (9-item version)
PHQ-15: Patient Health Questionnaire (15-item version)
PHR: Public Health Research
PMG: Programme Management Group
PPS: Persistent Physical Symptoms
PRINCE: Persistent physical symptoms Reduction Intervention: a system Change and Evaluation
PSC: Programme Steering Committee
RCT: Randomised Controlled Trial
REC: Research Ethics Committee
RfPB: Research for Patient
SD: Standard Deviation
TAU: Treatment as Usual
WSAS: Work & Social Adjustment Scale
