# Correction to: Integrated GP care for patients with persistent physical symptoms: feasibility cluster randomised trial

**DOI:** 10.1186/s12875-020-01302-x

**Published:** 2020-11-06

**Authors:** Meenal Patel, Kirsty James, Rona Moss-Morris, Mark Ashworth, Mujtaba Husain, Matthew Hotopf, Anthony S. David, Paul McCrone, Sabine Landau, Trudie Chalder, on behalf of the PRINCE Primary trial team

**Affiliations:** 1 Department of Psychological Medicine, Institute of Psychiatry, Psychology and Neuroscience, King’s College London, 16 De Crespigny Park, London, SE5 8AF UK; 2 Department of Biostatistics and Health Informatics, Institute of Psychiatry, Psychology and Neurosciences, Psychology and Neuroscience King’s College, London, UK; 3 Psychology Department, Institute of Psychiatry, Psychology and Neuroscience, King’s College, London, UK; 4 School of Population Health and Environmental Sciences, Faculty of Life Sciences and Medicine King’s College London, London, UK; 5 UK South London and Maudsley NHS Foundation Trust, London, UK; 6 Division of Psychiatry, Maple House, UCL Institute of Mental Health, 149 Tottenham Court Road, London, W1T 7NF UK; 7 Institute for Lifecourse Development, University of Greenwich, Old Royal Naval College, Park Row, Greenwich, London, SE10 9LS UK

**Correction to: BMC Fam Pract 21, 207 (2020)**

10.1186/s12875-020-01269-9

Following publication of the original article [1], an error which was reported in the title was corrected:

From: BMC family practice integrated GP care for patients with persistent physical symptoms: feasibility cluster randomised trial

To: Integrated GP care for patients with persistent physical symptoms: feasibility cluster randomised trial
